# Value of right heart haemodynamics for risk stratification of patients with pulmonary arterial hypertension at follow-up

**DOI:** 10.1093/eschf/xvaf012

**Published:** 2026-02-17

**Authors:** Laura Scelsi, Roberto Badagliacca, Michele D’Alto, Mauro Acquaro, Pietro Ameri, Paola Argiento, Natale Daniele Brunetti, Gavino Casu, Nadia Cedrone, Paola Confalonieri, Marco Corda, Michele Correale, Carlo D’Agostino, Elisabetta De Tommasi, Domenico Filomena, Giuseppe Galgano, Alessandra Greco, Massimo Grimaldi, Carlo Lombardi, Rosalinda Madonna, Giovanna Manzi, Valentina Mercurio, Alexandra Mihai, Massimiliano Mulè, Giuseppe Paciocco, Silvia Papa, Tommaso Recchioni, Antonella Romaniello, Emanuele Romeo, Davide Stolfo, Annalisa Turco, Marco Vatrano, Patrizio Vitulo, Carmine Dario Vizza, Stefano Ghio, The Italian Pulmonary Hypertension NETwork (iPHNET), Piergiuseppe Agostoni, Carlo Albera, Davide Barbisan, Vincenzo Bellomo, Marta Beretta, Marco Biolo, Federico Biondi, Andrea Bonelli, Renato Carignola, Francesco Cassadonte, Maria Alberta Cattabiani, Vincenzo Antonio Ciconte, Marco Confalonieri, Chiara Cresci, Alessandra Cuomo, Raffaele De Caterina, Gabriele Di Gesaro, Stefania Farina, Martino Fortunato, Giulia Gagno, Pietro Geri, Daniele Ghiraldin, Daniela Giannazzo, Giorgio Giardina, Maria Chiara Grimaldi, Ludovico Lanfranchi, Mariangela Lattanzio, Antonella Mannarini, Lavinia Martino, Beatrice Pezzuto, Francesca Rampini, Imma Romanazzi, Susanna Sciomer, Gianmarco Scoccia, Piermario Scuri, Corrado Tamburino, Lucia Tricarico, Sara Uras

**Affiliations:** UOC Cardiologia 1, Fondazione IRCCS Policlinico S Matteo, Pavia 27100, Italy; Department of Clinical, Anesthesiological and Cardiovascular Sciences—Sapienza University of Rome, Rome, Italy; Department of Cardiology, Monaldi Hospital—University “L. Vanvitelli”, Naples, Italy; UOC Cardiologia 1, Fondazione IRCCS Policlinico S Matteo, Pavia 27100, Italy; Cardiology Unit, IRCCS Ospedale Policlinico San Martino, Genova, Italy; Department of Internal Medicine, University of Genova, Genova, Italy; Department of Cardiology, Monaldi Hospital—University “L. Vanvitelli”, Naples, Italy; Department of Medical and Surgical Sciences, University of Foggia, Foggia, Italy; Azienda Ospedaliero Universitaria Sassari, UOC Cardiologia, Sassari, Italy; Unità di Medicina Interna, Ospedale S. Pertini, Rome, Italy; Pulmonology Unit, Heart-Thorax-Vessels Department, University Hospital of Cattinara, Trieste, Italy; Cardiology Unit, Azienda Ospedaliera “G. Brotzu” San Michele, Cagliari, Italy; Cardiology Department, Ospedali Riuniti University Hospital, Foggia, Italy; Cardiology Department, University Hospital Policlinico Consorziale Bari, Bari, Italy; Cardiology Department, University Hospital Policlinico Consorziale Bari, Bari, Italy; Department of Clinical, Anesthesiological and Cardiovascular Sciences—Sapienza University of Rome, Rome, Italy; Department of Cardiology, “F.Miulli” Hospital, Acquaviva delle Fonti, Bari, Italy; UOC Cardiologia 1, Fondazione IRCCS Policlinico S Matteo, Pavia 27100, Italy; Department of Cardiology, “F.Miulli” Hospital, Acquaviva delle Fonti, Bari, Italy; Cardiologia, Università degli studi di Brescia, Brescia, Italy; Cardiology Unit, Department of Surgical, Medical, Molecular Pathology and Critical Area Medicine, University of Pisa—UNIPI, Pisa, Italy; Department of Clinical, Anesthesiological and Cardiovascular Sciences—Sapienza University of Rome, Rome, Italy; Department of Translational Medical Sciences, Federico II University of Naples, Naples, Italy; Department of Clinical, Anesthesiological and Cardiovascular Sciences—Sapienza University of Rome, Rome, Italy; Cardiology Unit, IRCCS, Istituto Mediterraneo Trapianti e Terapie ad Alta Specializzazione (ISMETT), Palermo, Italy; Dipartimento Cardio-Toraco-Vascolare, Clinica Pneumologica, Azienda Ospedaliera San Gerardo, Monza, Italy; Department of Clinical, Anesthesiological and Cardiovascular Sciences—Sapienza University of Rome, Rome, Italy; Department of Clinical, Anesthesiological and Cardiovascular Sciences—Sapienza University of Rome, Rome, Italy; Cardiology Unit, S. Andrea Hospital, Rome, Italy; Department of Cardiology, Monaldi Hospital—University “L. Vanvitelli”, Naples, Italy; Cardiovascular Department, Azienda Sanitaria Universitaria Giuliano Isontina, Trieste, Italy; UOC Cardiologia 1, Fondazione IRCCS Policlinico S Matteo, Pavia 27100, Italy; UOC di Cardiologia—Azienda Ospedaliera “Pugliese-Ciaccio”, Catanzaro, Italy; Pulmonology Unit, IRCCS—Istituto Mediterraneo Trapianti e Terapie ad Alta Specializzazione (ISMETT), Palermo, Italy; Department of Clinical, Anesthesiological and Cardiovascular Sciences—Sapienza University of Rome, Rome, Italy; UOC Cardiologia 1, Fondazione IRCCS Policlinico S Matteo, Pavia 27100, Italy

**Keywords:** Pulmonary arterial hypertension, Prognostic markers, Right heart catheterization

## Abstract

**Introduction:**

ESC/ERS guidelines recommend risk stratification of prevalent patients with pulmonary arterial hypertension (PAH) using noninvasive parameters, whereas right heart haemodynamic parameters are left to the clinician’s discretion if deemed necessary. The study aimed to define the possible contribution of invasive haemodynamic parameters in predicting both the risk of death from all causes and the risk of clinical worsening (CW) in patients with PAH categorized at follow-up by the noninvasive ESC/ERS 4-strata risk stratification model.

**Methods:**

We evaluated incident patients with PAH enrolled in 11 Italian centres between 2005 and 2021 who had a first follow-up right heart catheterization within 6−12 months of diagnosis. In each noninvasive risk category, patients were subsequently stratified in a subgroup with a good haemodynamic profile if stroke volume index was ⩾38 mL/m^2^ and right atrial pressure was <8 mmHg and a subgroup with a poor haemodynamic profile if stroke volume index <38 ml/m^2^ and/or right atrial pressure ⩾8 mmHg. Median follow-up was 3.7 years (interquartile range 1.2–6.8) months.

**Results:**

Among low-risk patients (*n* = 162) survival was similar, but the CW rate was better in the good haemodynamic compared with the poor haemodynamic subgroup (*P* = .033). Among patients at intermediate-low risk (*n* = 240), both survival and CW rates were significantly better in the good haemodynamic subgroup compared with the poor haemodynamic subgroup (*P* = .028 and *P* = .011, respectively). Among patients at intermediate-high risk (*n* = 339), the CW rate was similar but survival was significantly better in the good haemodynamic than in the poor haemodynamic subgroup (*P* = .015). In the high-risk group, only 1 out of 28 patients had a good haemodynamic profile.

**Conclusion:**

In prevalent patients with PAH, a good haemodynamic profile predicts better survival in intermediate-risk patients and, importantly, a lower CW rate in low-risk patients.

## Graphical Abstract

**Graphical Abstract:**
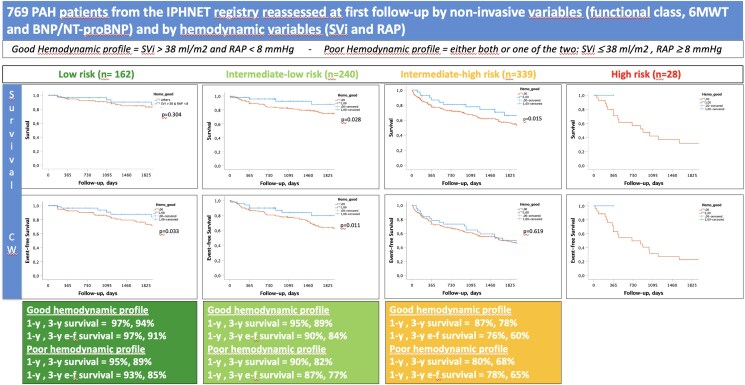
KM curves show survival rates and event free survival rates in 769 patients with PAH stratified at follow-up first by noninvasive variables and then by haemodynamic variables. In the high-risk group only 1 patient had a good haemodynamic profile and the second stratification step was not possible.

## Introduction

Pulmonary arterial hypertension (PAH) is a rare condition characterized by proliferation and remodelling of the small pulmonary arteries, leading to a progressive increase in pulmonary vascular resistance (PVR); ultimately, death may occur mainly due to right ventricular failure.^1^

Over the years, the importance of risk stratification has gained significant momentum in the field of PAH. The first tool for risk stratification of patients with PAH dates back to the early year 90’s, when a predictive equation based on haemodynamic data from the US National Institutes of Health registry was formulated.^1,2^ Since then, significant progress has been made in understanding the pathophysiological and clinical features of PAH, allowing for more comprehensive prognostic stratification and better care of these patients.^3–10^ Current guidelines recommend that approved PAH therapies should be initiated and adjusted during follow-up based on individual risk stratification.^11^

For several valid reasons, recent approaches to risk stratification have focused on simplicity, i.e. on the use of a reduced number of non-invasive parameters. The 2022 ESC/ERS guidelines support a 4 strata risk stratification model for patients with prevalent PAH, which is based on WHO functional class, brain natriuretic peptide (BNP) or N terminal-pro-BNP (NT-proBNP) plasma levels and distance walked in the 6 min walking test (6MWD). Right heart haemodynamic parameters are left to clinician’s discretion if deemed as necessary.^11^

However, not only when the decision-making process involves difficult options it may be necessary to have more accurate predictions than those achievable with non-invasive parameters. Low and intermediate-risk groups are in fact clinically heterogenous groups and many of the patients included in these groups may deteriorate rapidly and have poor outcomes.

Based on this background, the potential of haemodynamic parameters investigated by right heart catheterization (RHC) in supporting the risk stratification during follow-up needs to be reassessed. A re-analysis of the French registry recently showed that the haemodynamic variables at follow-up do not improve the risk stratification of patients with PAH classified at low or at high risk of death according to the non-invasive 4 strata model.^12^ On the contrary, among patients at intermediate-low or intermediate-high risk at follow-up, stroke volume index (SVi) and mixed venous oxygen saturation (SvO_2_) improved the prediction of transplant-free survival. A two-step approach based on noninvasive 4-strata model (first step) and right heart haemodynamics (second step) in patients at intermediate risk, provided incremental accuracy as compared with the noninvasive 4-strata model alone.^12^

The objective of the present study was to define the possible contribution of invasive haemodynamic assessment in predicting not only the risk of death from all causes but also the risk of clinical worsening (CW) in patients with PAH categorized at follow-up by the noninvasive ESC/ERS 4-strata risk stratification model. To this aim, we used the haemodynamic parameters and relative cut-off values recommended by the latest 2022 ESC/ERS PH guidelines.^11^

## Methods

### Population and study design

We retrospectively reviewed the medical records of adult patients with newly diagnosed PAH who were hospitalized between January 2005 and December 2021 in 11 centres of the Italian Pulmonary Hypertension NETwork (iPHNET). The data were collected retrospectively from the local databases that are used for the prospective follow-up of patients with PAH in these centres.^13^ The diagnostic work-up of PAH conformed to the ESC/ERS guidelines available at that time with the typical haemodynamic profile of pre-capillary pulmonary hypertension [defined by a mean pulmonary artery pressure (mPAP) ≥ 25 mmHg, a pulmonary artery wedge pressure (PAWP) ≤ 15 mmHg, and PVR ≥ 3 Wood Units]; the diagnostic algorithm included pulmonary function tests, lung perfusion scan, and computer tomography scan. Positive response to acute vasodilator testing with nitric oxide at the time of diagnosis was an exclusion criterion as well as age under 18 years; patients with portopulmonary hypertension or uncorrected congenital heart disease were also excluded. Living patients who underwent a first follow-up including RHC between 6 and 12 months after diagnosis were included in the study. All patients were followed for survival and for CW defined as death, any hospitalization for worsening PAH (including transplantation or initiation of parenteral prostanoids), a decrease of more than 15% from baseline in the 6MWD combined with WHO functional class III or IV symptoms.^14^ The study complied with the Declaration of Helsinki and was first approved by the Institutional Review Board for human studies of Policlinico Umberto I—Sapienza University of Rome (Protocol no. 638/18, coordinator center) and then by the local IRBs.

### Haemodynamic measurements

Right heart catheterization was performed in accordance with the European guidelines. The following parameters were measured or calculated: cardiac output (measured by thermodilution or by the Fick methods in the presence of severe tricuspid regurgitation), cardiac index (CI), stroke volume, stroke volume index (SVi), right atrial pressure (RAP), pulmonary artery pressure (PAP), PAWP, PVR, and pulmonary arterial compliance (PAC, calculated as stroke volume/pulse pressure). SvO_2_ was not routinely collected among all centres.

### Risk stratification

Risk assessment at follow-up was based on the simplified 4-strata risk-assessment tool recommended by the ESC/ERS guidelines for prevalent patients, which incorporates WHO functional class, 6MWD, NT-proBNP, or BNP plasma levels.

Right heart haemodynamics was used as a second step for refinement of risk assessment. In each non-invasive risk group, two subgroups were identified: a ‘good haemodynamic profile’ subgroup which included patients with both SVi ⩾38 mL·m2 and RAP <8 mmHg and a ‘poor haemodynamic profile’ subgroup, which included patients with at least one of the two: SVi <38 ml/m^2^ and/or RAP ⩾8 mmHg. In the present study, SVi and RAP were selected a priori because these parameters are indicated in the latest (as well as in the previous) PH Guidelines as robust and consistent prognostic parameters, and because these parameters have independent and additive pathophysiological significance, since SVi reflects right heart pump function and RAP systemic venous congestion.^1–3,11^ The prognostic cut-off points for SVi and RAP were those recommended by ESC/ERS guidelines for incident patients with PAH.^11^

### Statistical analysis

Continuous data are expressed as mean ± standard deviation or median and interquartile range (IQR), and categorical data are expressed as counts and proportions. Normality was assessed using the Kolmogorov–Smirnov test. Comparisons among risk profiles were made by using one-way analysis of variance (ANOVA) for variables normally distributed. If significant differences were found, post-hoc comparisons (Duncan’s multiple range test, Scheffé test) were used to determine the statistical significance among groups. The Kruskal–Wallis test was used for non-parametric comparison. Chi square or Fisher’s exact tests were used to analyse the categorical data. Event-free survival curves among groups were generated using the Kaplan–Meier method, and the log-rank test was used to evaluate differences between groups. The proportional-hazards assumption was tested using log-minus-log plots for categorical variables. All statistical analyses were performed using IBM SPSS statistical package (version 27.0) and open source package for R.

## Results

### Baseline characteristics of the patients

Between January 2005 and December 2021, 1015 incident patients with PAH, who were non-responsive to nitric oxide administration, were enrolled in the study. A total of 769 patients underwent a first follow-up including RHC between 6 and 12 months after diagnosis, and these patients formed the population of the present study. As shown in *Table 1*, the majority of patients had a diagnosis of idiopathic PAH, with a mean age of 59 years and mostly women. The 199 CTD patients were equally distributed across risk strata [39 patients (24%) in low-risk group; 59 patients (24.5%) in intermediate-low risk group; 94 patients (27.7%) in intermediate-high risk; 7 patients (25.0%) in high-risk group]. The majority patients were in WHO functional class III with severe pulmonary hypertension at RHC and impaired exercise capacity at 6 min walking test. The number of comorbidities was low (hyperlipidaemia *n* = 76 patients, diabetes *n* = 41 patients, thyroid disease *n* = 57 patients, systemic hypertension *n* = 131 patients, depression *n* = 51 patients) and analysing their distribution across the four risk strata, they were similar (*P* = ns for all). Atrial fibrillation was also unusual (*n* = 21 patients).

**Table 1:** Clinical and hemodynamic characteristics of patients according to their risk.

	All pts *N* = 769	Low risk *n* = 162	Int-low risk *N* = 240	Int-high risk *N* = 339	High risk *N* = 28	*P* (ANOVA)	*P* (post hoc)
**Age, years**	58.7 ± 14.3	54.3 ± 13.7	57.2 ± 14.6	61.7 ± 13.5	61.2 ± 16.6	.000	
**Female sex, n (%)**	494 (64,2)						
**BMI**	24.7 ± 4.4	24.7 ± 4.4	24.9 ± 4.5	23.8 ± 3.9	26.1 ± 7.4	.845	
**PAH type, n (%)**							
Idiopathic	469 (61%)						
Heritable	31 (4%)						
Drug induced	11 (1%)						
CTD	199 (26%)						
HIV	59 (8%)						
**WHO class, n (%)**							
I	162 (21)	16	16	0	0		
II	240 (31)	146	166	5	0		
III	339 (44)	0	58	333	9		
IV	28 (4)	0	0	1	19	global *P* = .000	
**6MWT, m**	375.0 ± 118.3	496 ± 54	407 ± 66	315 ± 66	120 ± 77	.000	
**NT-proBNP, pg/ml**	85.5 ± 559.0	55 ± 120	53 ± 168	313 ± 801	503 ± 602	.359	
**Haemodynamics**							
RAP, mmHg	7.7 ± 4.7	6.4 ± 3.9	7.9 ± 4.7	8.0 ± 4.9	11.2 ± 6.4	.000	1 vs 2: .008 1 vs 3: .001 1 vs 4: .000 2 vs 3: 1.000 2 vs 4: .002 3 vs 4: .002
mPAP, mmHg	44.1 ± 15.9	40.0 ± 15.7	43.1 ± 16,6	46.3 ± 15.0	50.7 ± 15.8	.000	
CI, l/min/m^2^	2.7 ± 0.7	3.0 ± 0.7	2.8 ± 0.7	2.5 ± 0.6	1.9 ± 0.4	.000	1 vs 2: .016 1 vs 3: .000 1 vs 4: .000 2 vs 3: .000 2 vs 4: .000 3 vs 4: .000
SVi, ml/m^2^/beat	35.2 ± 9.9	40.2 ± 10.7	36.2 ± 9.8	32.8 ± 8.2	24.4 ± 6.9	.000	1 vs 2: .000 1 vs 3: .000 1 vs 4: .000 2 vs 3: .000 2 vs 4: .000 3 vs 4: .000
PAWP, mmHg	9.4 ± 3.6	9.5 ± 3.3	9.3 ± 3.5	9.2 ± 3.7	9.4 ± 3.3	.017	All NS
PVR, WU	8.7 ± 5.5	6.8 ± 4.0	8.0 ± 5.5	9.7 ± 5.5	14.0 ± 8.1	.000	1 vs 2: .245 1 vs 3: .000 1 vs 4: .000 2 vs 3: .001 2 vs 4: .000 3 vs 4: .001
PAC, mmHg/ml	1.8 ± 1.2	2.4 ± 1.7	1.7 ± 1.1	1.5 ± 0.9	1.0 ± 0.8	.000	1 vs 2: .000 1 vs 3: .000 1 vs 4: .000 2 vs 3: .366 2 vs 4: .132 3 vs 4: .691
HR, beats/min	76.2 ± 9.7	74.5 ± 8.3	77.3 ± 10.2	76.2 ± 9.7	77.9 ± 10.0	.029	1 vs 2: .027 1 vs 3: .396 1 vs 4: .472 2 vs 3: 1.000 2 vs 4: 1.000 3 vs 4: 1.000

BMI, body mass index; CI, cardiac index; HR, heart rate; mPAP, mean pulmonary artery pressure; PAC, pulmonary artery compliance; PAH, pulmonary arterial hypertension; PAWP, pulmonary artery wedge pressure; PVR, pulmonary vascular resistance; RAP, right atrial pressure; SVi, stroke volume index.

The most frequently used treatment strategy within the first 6–12 months after PAH diagnosis was monotherapy (69%), followed by dual or triple oral combination therapy (22%) and triple combination therapy including parenteral prostanoids (9%). Oral monotherapy was more frequent among patients in the low-risk strata and less common in the high-risk strata [132 patients (81%) in low-risk groups; 176 patients (73%) in intermediate-low risk group; 210 patients (62%) in intermediate-high risk group; 13 patients (46%) in high risk group].

*Table 1* summarizes the patients’ characteristics and haemodynamic parameters according to non-invasive risk groups at first follow-up. The number of missing data were unlikely to bias statistical analysis as they were <5% of the total amount of data collected and <10% for each single variable (27 patients had both 6MWD and NT-proBNP data missing, while 19 patients had 6MWD or NT-proBNP missing). According to the ESC/ERS 4-strata risk assessment tool, 162 (21.1%) patients were at low risk, 240 (31.2%) at intermediate-low risk, 339 (44.1%) were at intermediate-high risk and 28 (3.5%) at high risk. As expected, the low-risk group included patients who were asymptomatic or mildly symptomatic, had normal NT-proBNP plasma levels and a normal exercise capacity. The intermediate-low risk group included patients with normal NT-proBNP plasma levels, who were slightly more symptomatic and had poorer exercise capacity as compared to low-risk patients. The intermediate-high risk and high-risk groups included symptomatic patients with high NT-proBNP levels and very poor exercise capacity. Right heart haemodynamic parameters tracked closely with non-invasive risk groups, since all parameters progressively deteriorated from low-risk to high-risk group. Of notice, there was a significant proportion of low-risk patients who had elevated PVR values: the median value in the low-risk group approached 7 WU (*Table 1*). In addition, in the low-risk group, the percentage of patients with SVi <38 mL/m^2^ was 48.1% and the percentage of patients with RAP ⩾8 mmHg was 29.6% (*Table 2*). The percentage of patients with abnormal haemodynamic values was progressively higher in the intermediate-low and intermediate-high risk groups; in the high-risk group nearly 93% of patients had a low SVi and nearly 70% had an abnormal RAP value.

**Table 2:** Patients with abnormal haemodynamic values

	All pts *N* = 769	Low risk *n* = 162	Int-low risk *N* = 240	Int-high risk *N* = 339	High risk *N* = 28	*P* (ANOVA)
RAP ⩾8 mmHg (%)	44.4	29.6	44.6	49.5	67.9	.000
SVi <38 mL/m^2^ (%)	66.7	48.1	62.5	76.4	92.9	.000
‘Good haemodynamic’ profile	20.6	36.5	21.2	13.0	3.5	.000
‘Poor haemodynamic’ profile	79.4	63.5	78.8	87.0	96.5	.000

RAP, right atrial pressure; SVi, stroke volume index.

### Prognosis according to non-invasive risk and haemodynamic refinements

The median follow-up period was 3.7 years (IQR 1.2–6.8) months. During follow-up, 27 patients died (16.6%) and 39 patients (24.1%) had a CW episode in the low-risk group. In the intermediate-low risk group, 56 patients died (23.3%) and 90 patients (37.5%) experienced clinical worsening during follow-up. In the intermediate-high risk group, 142 patients died (41.9%) and 190 patients (56.0%) experienced clinical worsening during follow-up. In the high-risk group, 16 patients died (57.2%) and 21 patients (75%) experienced clinical worsening during follow-up.

In the low-risk group, survival was similar, but CW rate was significantly better in the subgroup with good haemodynamic profile (*P* = .033). In this group, 1-y and 3-y survival were 97% and 94% in patients with a good haemodynamic profile vs 95% and 89% in patients with a poor haemodynamic profile; 1-y and 3-y CW-free rate were 97% and 91% in patients with a good haemodynamic profile vs 93% and 85% in patients with a poor haemodynamic profile.

In the intermediate-low risk group, both survival (*P* = .028) and CW-free rate (0.011) were significantly better in the subgroup with good haemodynamic profile. In this group, 1-y and 3-y survival were 95% and 89% in patients with a good haemodynamic profile vs 90% and 82% in patients with a poor haemodynamic profile; 1-y and 3-y CW-free rate were 90% and 84% in patients with a good haemodynamic profile vs 87% and 77% in patients with a poor haemodynamic profile.

In the intermediate-high risk group survival was better (*P* = .015) but CW-free rate was similar (*P* = .619) in the subgroup with good haemodynamic profile. In this group, 1-y and 3-y survival were 87% and 78% in patients with a good haemodynamic profile vs 80% and 68% in patients with a poor haemodynamic profile; 1-y and 3-y CW-free rate were 76% and 60% in patients with a good haemodynamic profile vs 78% and 65% in patients with a poor haemodynamic profile. In the high-risk group, all but 1 patient had a poor haemodynamic profile.

## Discussion

There are two main findings of this Italian multicentre cohort study. First, the study confirms that haemodynamic variables improve the risk stratification at follow-up of patients with PAH classified at intermediate-low or intermediate-high risk according to the noninvasive 4-strata model, as recently shown in the French registry.^12^ Second, as an emerging concept, the study also demonstrates that the haemodynamic profile can be important to refine the prognosis also in low-risk patients, since a good haemodynamic profile at follow-up predicts a lower CW rate at 1 and 3 years.

The importance of refining risk stratification at follow-up in patients with PAH classified into intermediate groups according to the European noninvasive tool is evident simply by considering the number of such patients. Intermediate-risk patients together represent approximately two-thirds of all prevalent patients (indeed, these two groups included 63% of patients in the French cohort and 75% of all patients in the current Italian cohort). According to the results presented here, therefore, there are many patients in whom the risk of death and the risk of CW could be more precisely gauged by performing RHC. This has recently been highlighted by Boucly *et al*.,^12^ who showed that the combination of SVi and SvO_2_ was able to identify a subgroup of patients at higher risk of mortality within both the ESC/ERS intermediate-low and intermediate-high risk. SVi and SvO_2_ emerged in the French cohort as the two most relevant independently prognostic haemodynamic variables. In the present study, SVi and RAP were selected a priori as robust and consistent prognostic parameters, with independent and additive pathophysiological significance, since SVi reflects right cardiac pump function and RAP reflects systemic venous congestion. In PAH, right ventricle (RV) to pulmonary artery coupling progresses to uncoupling through an increase in RV filling pressure (i.e. RAP) and a decrease in SVi, ultimately leading to heart failure.

The second interesting aspect of the present study is the demonstration that the haemodynamic profile can help predict the risk of clinical worsening in patients with PAH. This is a novel and important result of our study and deserves attention, since morbidity (not mortality) is very sensitive to PAH-specific treatments, as repeatedly demonstrated in event-driven PAH trials,^14–17^ as well as in observational studies.^18–21^ The association between haemodynamic profile and CW rate appeared consistent across the risk strata, from low- to intermediate-high risk strata, more than the link between haemodynamic profile and survival, which was limited to more advanced stages of the disease (intermediate-low and intermediate-high risk strata).

In the present study, 20% of patients at intermediate-low risk (*n* = 32) had a good haemodynamic profile and 1-y survival (95%) comparable with 1-y survival of patients classified at low risk (97%); 87% of patients at intermediate-high risk (*n* = 295) had a poor haemodynamic profile and 1-y survival rate (80%) similar to 1-y survival of patients classified at high risk (80%).

Most importantly, we should not underestimate the practical importance of using haemodynamic parameters to discriminate the risk of CW in patients who are classified at low risk according to non-invasive criteria. Patients at non-invasive low risk may worsen at follow-up despite PAH-specific treatments. The recent TORREY trial showed that the placebo cohort, including 60% of low-risk patients with PAH (REVEAL 2.0 risk score < 6), experienced disease progression within 24 weeks, resulting in a significant increase in NT-proBNP and right atrial area, and a decrease in the RV-free wall strain.^22^ The reason behind disease progression in patients at low risk may be that a substantial proportion of low-risk patients have an abnormal right heart haemodynamic profile. This hypothesis is suggested by the results of the present study: nearly two thirds of low-risk patients in the present series had either a low SVi or an elevated RAP. Thus, although all-cause death was similar in low-risk patients with a good or a poor haemodynamic profile, RHC remains critical for identifying those at the highest risk for future clinical worsening events, therefore, providing clinicians with the opportunity to plan a rigorous follow-up, not to delay the escalation of efficacious targeted PAH treatment.

### Study limitations

The main limitations of the study are its retrospective nature and the long enrolment period in the registry, which extends over 15 years, during which new drugs became available and the treatment strategy for patients with PAH changed from a sequential to an upfront combination approach; the proportion of patients in combination therapy may in fact be considered low. These conditions preclude the possibility of interpreting the role played by therapies in achieving (or not achieving) the desired goal of a low-risk state; however, they do not change the relationship between clinical/haemodynamic variables and prognosis. Second, although the threshold values used for the definition of a good or poor haemodynamic profile are based on the ESC/ERS guidelines definitions of SVi and RAP low-risk values, they remain somewhat arbitrary. This limitation might partially explain why the association between haemodynamic profile and events was different across risk strata; choosing different haemodynamic thresholds might have been particularly useful for risk stratification of high-risk patients. Although SVi and RAP cut-off points have been used with a *post hoc* validation strategy, based on previous ESC/ERS recommended values rather than from a new statistical analysis, the pathophysiology behind the use of SVi and RAP is robust: a ‘good’ RV function is in fact best identified when the right ventricle is able to maintain a normal pump function (i.e. normal SVi) without the necessity of using the compensatory mechanism of an increase in pre-load (i.e. normal RAP).

We have not further investigated the potential prognostic role of other haemodynamic parameters; this is another interesting issue because it has recently been shown that several haemodynamic parameters reflecting both RV afterload and RV are prognostically relevant at first follow-up after starting targeted PAH treatment, and most of these are not yet defined in current ESC/ERS guidelines risk tables.^23^ Notably, the improved outcome after sotatercept was associated with an improvement in pulmonary artery pressure rather than with an improvement in SVi.^24–26^ All these issues deserve to be tested in future large multicentre studies. Finally, whether low-risk patients with poor right heart haemodynamic profile would benefit from early add-on treatments deserves dedicated, randomized, prospective studies.

## Conclusions

Repeating RHC within 6–12 months after diagnosis may help clinicians in stratifying the risk of patients with PAH at the first follow-up after diagnosis.

Haemodynamic parameters reflecting RV function and systemic venous congestion have an additional value compared to noninvasive parameters, since abnormal SVi and/or RAP allow to identify, among patients classified at intermediate risk, those with a poorer survival as well as, among patients classified at low risk, those with a higher rate of clinical worsening.

## Supplementary Material

xvaf012_Supplementary_Data

## Data Availability

Any additional data underlying this article will be shared on reasonable request to the corresponding author.

## Pre-registered Clinical Trial Number

None supplied.

## Appendix

**The Italian Pulmonary Hypertension NETwork (iPHNET) group further consists of:**

Piergiuseppe Agostoni, Cardiologia Clinica e Cardiologia Riabilitativa Centro Cardiologico Monzino IRCCS, Milano

Carlo Albera, SC Pneumologia U Ospedale Molinette Torino

Davide Barbisan, Azienda Sanitaria Universitaria Integrata of Trieste (ASUITS), University of Trieste Cardiovascular Department—Cattinara Hospital, Trieste, Italy

Vincenzo Bellomo, Ecclesiastical Entity General Regional Hospital Francesco Miulli, 161123, Department of cardiology, Acquaviva delle Fonti, Italy

Marta Beretta, Pulmonology Unit, IRCCS—Istituto Mediterraneo Trapianti e Terapie ad Alta Specializzazione (ISMETT), Palermo, Italy

Marco Biolo, Pulmonology Unit, Heart-Thorax-Vessels Dept., University Hospital of Cattinara, Trieste, Italy

Federico Biondi, Cardiovascular Department, Azienda Sanitaria Universitaria Giuliano Isontina, Trieste, Italy

Andrea Bonelli, Department of Medical and Surgical Specialties, Radiological Sciences, and Public Health, University and Civil Hospital, Brescia, Italy

Renato Carignola, SCDU Malattie dell’Apparato Respiratorio 2 AOU San Luigi Gonzaga, Orbassano, Torino, Italy

Francesco Cassadonte, Cardiology Unit, Hospital “Pugliese-Ciaccio” of Catanzaro, Italy

Maria Alberta Cattabiani, UO Cardiologia AOU di Parma—Ospedale maggiore di Parma, Italy

Vincenzo Antonio Ciconte, Cardiology Unit, Hospital “Pugliese-Ciaccio” of Catanzaro, Italy

Marco Confalonieri, Pulmonology Unit, Heart-Thorax-Vessels Dept., University Hospital of Cattinara, Trieste, Italy

Chiara Cresci, SO Pneumologia e Fisiopatologia Toraco-Polmonare AOU Careggi, Firenze

Alessandra Cuomo, Department of Translational Medical Sciences—Federico II University of Naples, Italy

Raffaele De Caterina, UOC Cardiologia 1 AOU Piasana, Pisa, Italy

Gabriele Di Gesaro, Pulmonology Unit, IRCCS—Istituto Mediterraneo Trapianti e Terapie ad Alta Specializzazione (ISMETT), Palermo, Italy

Stefania Farina, Cardiologia Clinica e Cardiologia Riabilitativa Centro cardiologico Monzino IRCCS, Milano

Martino Fortunato, Cardiology Department, Ospedali Riuniti University Hospital, Foggia, Italy

Giulia Gagno, Azienda Sanitaria Universitaria Integrata of Trieste (ASUITS), University of Trieste Cardiovascular Department—Cattinara Hospital

Pietro Geri, Pulmonology Unit, Heart-Thorax-Vessels Dept., University Hospital of Cattinara, Trieste, Italy

Daniele Ghiraldin, Department of Medical and Surgical Specialties, Radiological Sciences, and Public Health, University and Civil Hospital, Brescia, Italy

Daniela Giannazzo, Dipartimento Cardio-Toraco-Vascolare AOU Policlinico Vittorio Emanuele Presidio Ferrarotto, Catania

Giorgio Giardina, Cardiology Unit, Azienda Ospedaliera “G. Brotzu” San Michele, Cagliari, Italy

Maria Chiara Grimaldi, Cardiology Unit, S. Andrea Hospital, Rome, Italy

Ludovico Lanfranchi, Dipartimento Cardio-Toraco-Vascolare—Clinica Pneumologica AO San Gerardo, Monza, Italy

Mariangela Lattanzio, Cardiologia Ospedale di Circolo e Fondazione Macchi, Varese, Italy

Antonella Mannarini, Cardiology Department—University Hospital Policlinico Consorziale Bari, Italy

Lavinia Martino, Pulmonology Unit, IRCCS—Istituto Mediterraneo Trapianti e Terapie ad Alta Specializzazione (ISMETT), Palermo, Italy

Beatrice Pezzuto, Cardiologia Clinica e Cardiologia Riabilitativa Centro Cardiologico Monzino IRCCS, Milano

Francesca Rampini, Dept. of Cardiovascular and Respiratory Sciences—Sapienza University of Rome, Italy

Imma Romanazzi, Dipartimento Cardio-Toraco-Vascolare AOU Policlinico Vittorio Emanuele –Presidio Ferrarotto, Catania Italy

Susanna Sciomer, Dept. of Cardiovascular and Respiratory Sciences—Sapienza University of Rome, Italy

Gianmarco Scoccia, Dept. of Cardiovascular and Respiratory Sciences—Sapienza University of Rome, Italy

Piermario Scuri, Unità di Cardiologia Ospedale Papa Giovanni XXIII Centro Ospedaliero—24129 Bergamo

Corrado Tamburino, Dipartimento Cardio-Toraco-Vascolare AOU Policlinico Vittorio Emanuele –Presidio Ferrarotto, Catania, Italy

Lucia Tricarico, Cardiology Department, Ospedali Riuniti University Hospital, Foggia, Italy,

Sara Uras, ATS Sardegna-ASSL Nuoro, San Francesco Hospital Nuoro, Italy,

## References

[1] D'Alonzo GE, Barst RJ, Ayres SM, Bergofsky EH, Brundage BH, Detre KM, et al Survival in patients with primary pulmonary hypertension. Results from a national prospective registry. Ann Intern Med 1991;115:343–9. 10.7326/0003-4819-115-5-343 1863023

[2] Sandoval J, Bauerle O, Palomar A, Gomez A, Martínez-Guerra ML, Beltrán M, et al Survival in primary pulmonary hypertension validation of a prognostic equation. Circulation 1994;89:1733–44. 10.1161/01.CIR.89.4.1733 8149539

[3] Weatherald J, Boucly A, Chemla D, Savale L, Peng M, Jevnikar M, et al Prognostic value of follow-up hemodynamic variables after initial management in pulmonary arterial hypertension. Circ 2018;137:693–704. 10.1161/CIRCULATIONAHA.117.029254 29070502

[4] Boucly A, Weatherald J, Savale L, Jaïs X, Cottin V, Prevot G, et al Risk assessment, prognosis and guideline implementation in pulmonary arterial hypertension. Eur Respir J 2017;50:1700889. 10.1183/13993003.00889-2017 28775050

[5] Benza RL, Kanwar MK, Raina A, Scott JV, Zhao CL, Selej M, et al Development and validation of an abridged version of the REVEAL 2.0 risk score calculator, REVEAL Lite 2, for use in patients with pulmonary arterial hypertension. Chest 2021;159:337–46. 10.1016/j.chest.2020.08.2069 32882243 PMC7462639

[6] Benza RL, Miller DP, Gomberg-Maitland M, Frantz RP, Foreman AJ, Coffey CS, et al Predicting survival in pulmonary arterial hypertension: insights from the registry to evaluate early and long-term pulmonary arterial hypertension disease management (REVEAL). Circulation 2010;122:164–72. 10.1161/CIRCULATIONAHA.109.898122 20585012

[7] Hoeper MM, Kramer T, Pan Z, Eichstaedt CA, Spiesshoefer J, Benjamin N, et al Mortality in pulmonary arterial hypertension: prediction by the 2015 European pulmonary hypertension guidelines risk stratification model. Eur Respir J 2017;50:1700740. 10.1183/13993003.00740-2017 28775047

[8] Kylhammar D, Kjellström B, Hjalmarsson C, Jansson K, Nisell M, Söderberg S, et al A comprehensive risk stratification at early follow-up determines prognosis in pulmonary arterial hypertension. Eur Heart J 2018;39:4175–81. 10.1093/eurheartj/ehx257 28575277

[9] Hoeper MM, Pausch C, Olsson KM, Huscher D, Pittrow D, Grünig E, et al COMPERA 2.0: a refined four-stratum risk assessment model for pulmonary arterial hypertension. Eur Respir J 2022;60:2102311. 10.1183/13993003.02311-2021 34737226 PMC9260123

[10] Boucly A, Weatherald J, Savale L, de Groote P, Cottin V, Prévot G, et al External validation of a refined four-stratum risk assessment score from the French pulmonary hypertension registry. Eur Respir J 2022;59:2102419. 10.1183/13993003.02419-2021 34737227 PMC9245192

[11] Humbert M, Kovacs G, Hoeper MM, Badagliacca R, Berger RMF, Brida M, et al 2022 ESC/ERS Guidelines for the diagnosis and treatment of pulmonary hypertension. Eur Respir J 2023;61:2200879. 10.1183/13993003.00879-2022 36028254

[12] Boucly A, Beurnier A, Turquier S, Jevnikar M, de Groote P, Chaouat A, et al Risk stratification refinements with inclusion of haemodynamic variables at follow-up in patients with pulmonary arterial hypertension. Eur Respir J 2024;64:2400197. 10.1183/13993003.00197-2024 38663975 PMC11375514

[13] Poscia R, Ghio S, D'Alto M, Vitulo P, Mulè M, Albera C, et al ‘Real-life’ information on pulmonary arterial hypertension: the iPHnet project. Curr Med Res Opin 2014;30:2409–14. 10.1185/03007995.2014.960514 25180610

[14] Galiè N, Barberà JA, Frost AE, Ghofrani H-A, Hoeper MM, McLaughlin VV, et al Initial use of ambrisentan plus tadalafil in pulmonary arterial hypertension. N Engl J Med 2015;373:834–44. 10.1056/NEJMoa1413687 26308684

[15] Sitbon O, Channick R, Chin KM, Frey A, Gaine S, Galiè N, et al Selexipag for the treatment of pulmonary arterial hypertension. N Engl J Med 2015;373:2522–33. 10.1056/NEJMoa1503184 26699168

[16] Pulido T, Adzerikho I, Channick RN, Delcroix M, Galiè N, Ghofrani H-A, et al Macitentan and morbidity and mortality in pulmonary arterial hypertension. N Engl J Med 2013;369:809–18. 10.1056/NEJMoa1213917 23984728

[17] McLaughlin VV, Hoeper MM, Channick RN, Chin KM, Delcroix M, Gaine S, et al Pulmonary arterial hypertension-related morbidity is prognostic for mortality. J Am Coll Cardiol 2018;71:752–63. 10.1016/j.jacc.2017.12.010 29447737

[18] Badagliacca R, Ghio S, D'Alto M, Ameri P, Correale M, Filomena D, et al Relevance of echocardiography-derived phenotyping in patients with pulmonary arterial hypertension treated with initial oral combination therapy: an Italian Pulmonary Hypertension Network (iPHNET) study. Am J Respir Crit Care Med 2024;210:362–5. 10.1164/rccm.202402-0431LE 38820124

[19] Ghio S, Badagliacca R, D'Alto M, Scelsi L, Argiento P, Brunetti ND, et al Right ventricular phenotyping in incident patients with idiopathic pulmonary arterial hypertension. J Heart Lung Transplant 2024;43:1668–76. 10.1016/j.healun.2024.06.003 38942159

[20] D'Alto M, Badagliacca R, Argiento P, Romeo E, Farro A, Papa S, et al Risk reduction and right heart reverse remodeling by upfront triple combination therapy in pulmonary arterial hypertension. Chest 2020;57:376–83. 10.1016/j.chest.2019.09.009 31563498

[21] D'Alto M, Badagliacca R, Lo Giudice F, Argiento P, Casu G, Corda M, et al Hemodynamics and risk assessment 2 years after the initiation of upfront ambrisentan–tadalafil in pulmonary arterial hypertension. J Heart Lung Transplant 2020;39:1389–97. 10.1016/j.healun.2020.08.016 32933828

[22] Frantz RP, McLaughlin VV, Sahay S, Escribano Subías P, Zolty RL, Benza RL, et al Seralutinib in adults with pulmonary arterial hypertension (TORREY): a randomised, double-blind, placebo-controlled phase 2 trial. Lancet Respir Med 2024;12:523–34. 10.1016/S2213-2600(24)00072-9 38705167

[23] Dardi F, Guarino D, Ballerini A, Bertozzi R, Donato F, Cennerazzo F, et al Prognostic role of haemodynamics at follow-up in patients with pulmonary arterial hypertension: a challenge to current European Society of Cardiology/European Respiratory Society risk tools. ERJ Open Res 2024;10:00225-2024. 10.1183/23120541.00225-2024 39104950 PMC11298999

[24] Humbert M, McLaughlin V, Gibbs JSR, Gomberg-Maitland M, Hoeper MM, Preston IR, et al Sotatercept for the treatment of pulmonary arterial hypertension. N Engl J Med 2021;384:1204–15. 10.1056/NEJMoa2024277 33789009

[25] Hoeper MM, Badesch DB, Ghofrani HA, Gibbs JSR, Gomberg-Maitland M, McLaughlin VV, et al Phase 3 trial of sotatercept for treatment of pulmonary arterial hypertension. N Engl J Med 2023;388:1478–90. 10.1056/NEJMoa2213558 36877098

[26] Souza R, Badesch DB, Ghofrani HA, Gibbs JSR, Gomberg-Maitland M, McLaughlin VV, et al Effects of sotatercept on haemodynamics and right heart function: analysis of the STELLAR trial. Eur Respir J 2023;62:2301107. 10.1183/13993003.01107-2023 37696565 PMC10512088

