# Supplementary material for: Value of right heart haemodynamics for risk stratification of patients with pulmonary arterial hypertension at follow-up
Source: ESC Heart Fail. 2026 Feb 17;13(1):xvaf012. doi: 10.1093/eschf/xvaf012 (PMC13108289; doi:10.1093/eschf/xvaf012)
Supplement: xvaf012_Supplementary_Data [file xvaf012_supplementary_data.zip › Supplementary Figure captions.docx]

**Supplementary Figure 1**

Survival (A) and event free survival (B) in low-risk group according to ESC/ERS guidelines 4-strata tool, categorized according to hemodynamic variables: good hemodynamic profile (blue line), poor hemodynamic profile (red line).

**Supplementary Figure 2**

Survival (A) and event free survival (B) in intermediate-low risk group according to ESC/ERS guidelines 4-strata tool, categorized according to hemodynamic variables: good hemodynamic profile (blue line), poor hemodynamic profile (red line).

**Supplementary Figure 3**

Survival (A) and event free survival (B) in intermediate-high risk group according to ESC/ERS guidelines 4-strata tool, categorized according to hemodynamic variables: good hemodynamic profile (blue line), poor hemodynamic profile (red line).

**Supplementary Figure 4**

In the high-risk group according to ESC/ERS guidelines 4-strata tool, only 1 patient had a good hemodynamic profile and no further hemodynamic stratification was possible.
