# Supplementary figures and images for: Value of right heart haemodynamics for risk stratification of patients with pulmonary arterial hypertension at follow-up
Source: ESC Heart Fail. 2026 Feb 17;13(1):xvaf012. doi: 10.1093/eschf/xvaf012 (PMC13108289; doi:10.1093/eschf/xvaf012)

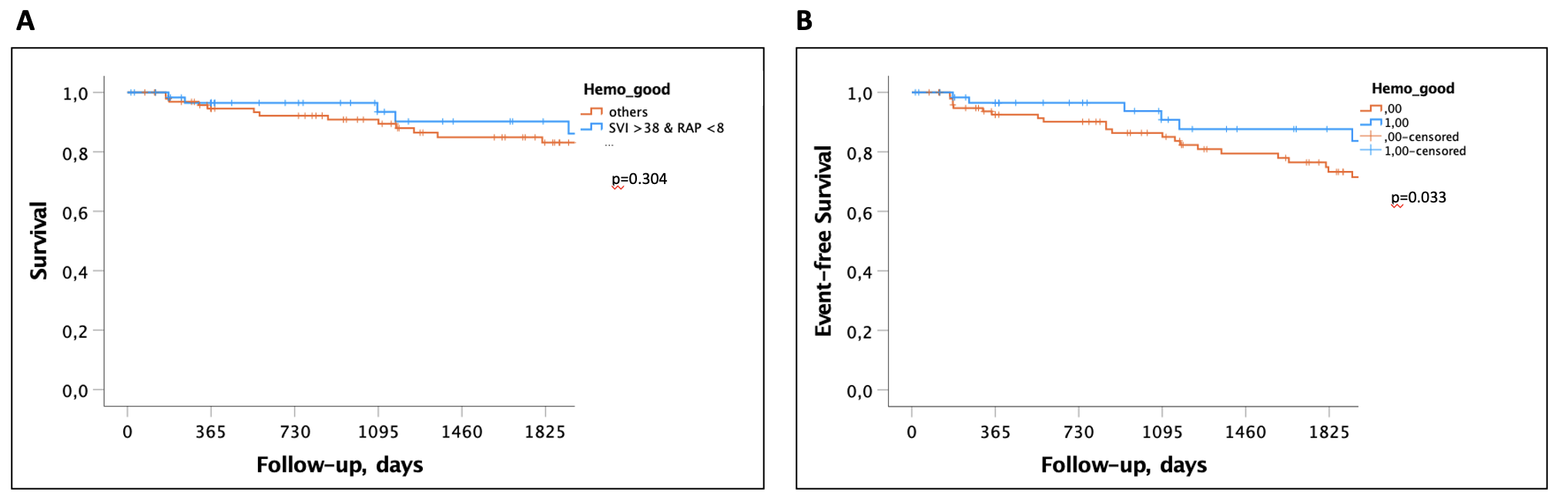

Supplement: xvaf012_Supplementary_Data [file xvaf012_supplementary_data.zip › suppl fig 1.png]

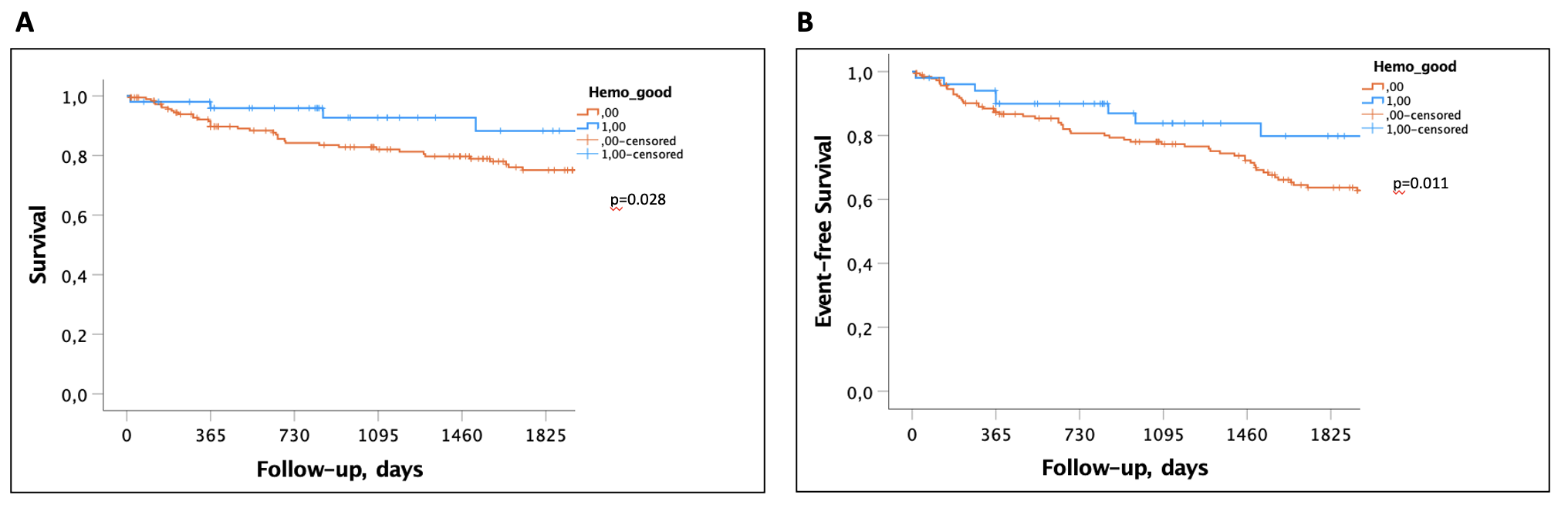

Supplement: xvaf012_Supplementary_Data [file xvaf012_supplementary_data.zip › suppl fig 2.png]

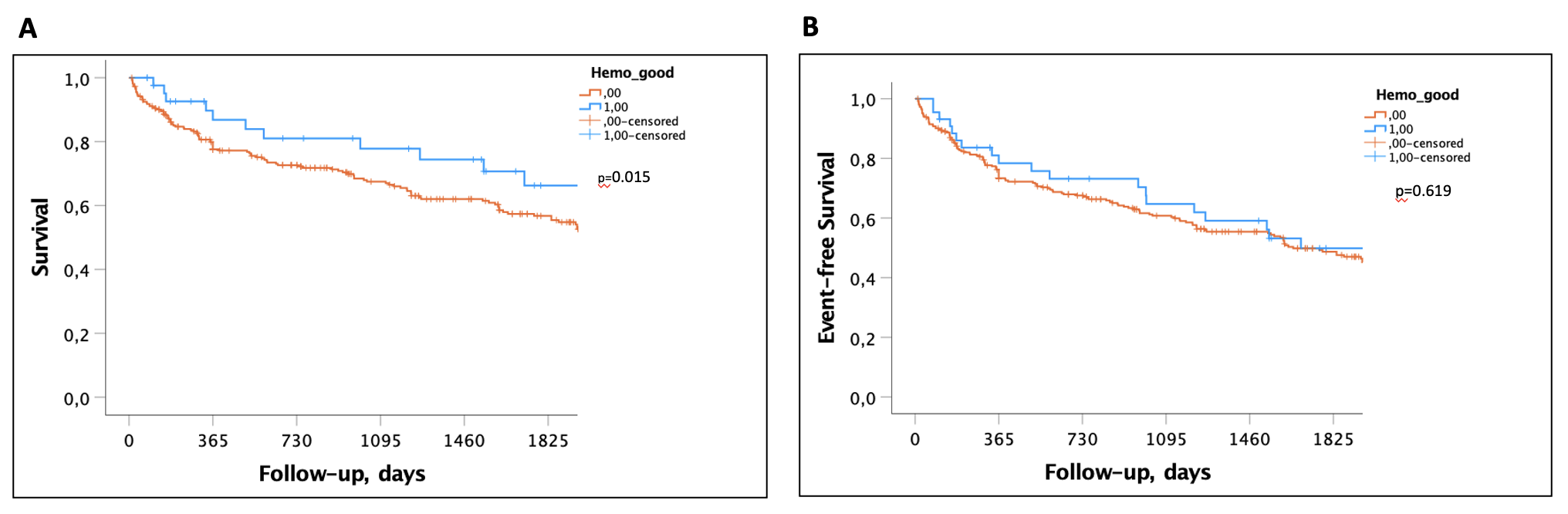

Supplement: xvaf012_Supplementary_Data [file xvaf012_supplementary_data.zip › suppl fig 3.png]

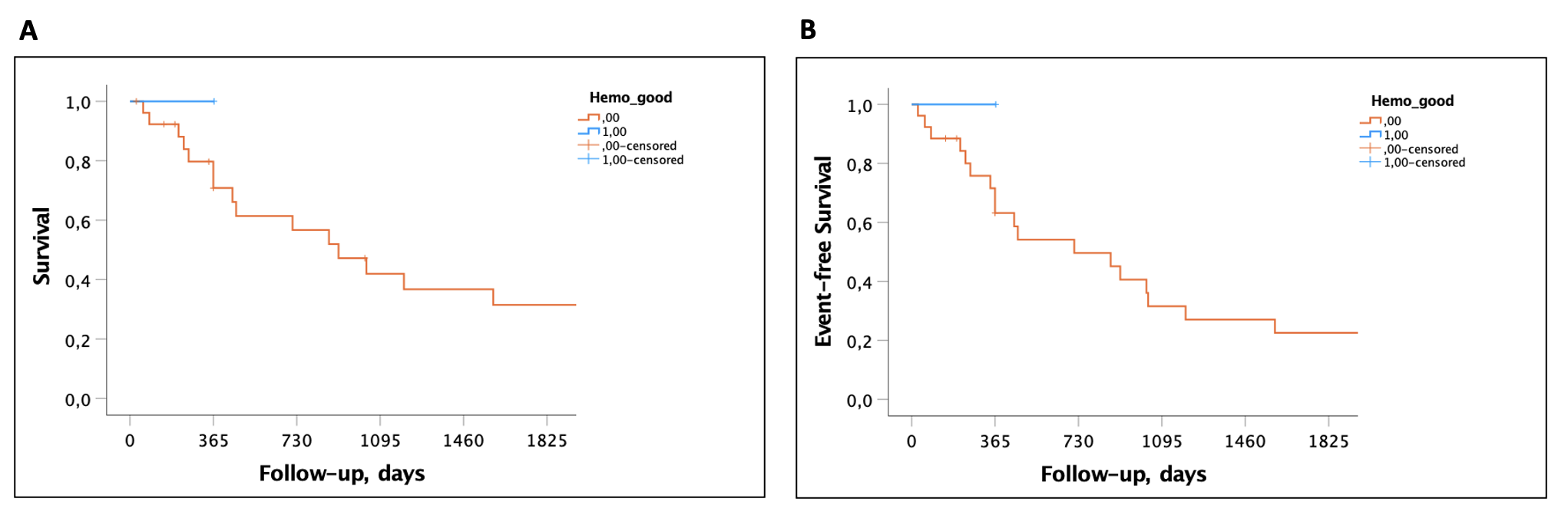

Supplement: xvaf012_Supplementary_Data [file xvaf012_supplementary_data.zip › suppl fig 4.png]
